# Editorial: Skateboarding and society: intersections, influences, and implications

**DOI:** 10.3389/fspor.2025.1649479

**Published:** 2025-07-03

**Authors:** Bethany Geckle, Kevin Fang, Jorge Ricardo Saraví

**Affiliations:** ^1^ Department of Sport Leadership and Management, Miami University, Oxford, OH, United States; ^2^ Department of Geography Environment and Planning, Sonoma State University, Rohnert Park, CA, United States; ^3^ Departamento de Educación Física, Universidad Nacional de La Plata Facultad de Humanidades y Ciencias de la Educacion, La Plata, Argentina

**Keywords:** skateboarding, urban development, social development, inclusion, exclusion, placemaking

**Editorial on the Research Topic**
Skateboarding and society: intersections, influences, and implications

Over the last 75 years, skateboarding has come a long way, evolving from a niche activity among surfers in California to a global phenomenon with millions of participants worldwide. Throughout its history, the activity of skateboarding has diversified. The people who skateboard, where they ride, how they ride, and their reasons for riding have evolved as skateboarders have developed new and creative ways to participate in the activity. As the activity of skateboarding has changed, so too have skateboarders’ interactions with their surrounding communities, both physically and socially. These unique characteristics of skateboarding have attracted the attention of researchers over the last several decades. Books, theses, and articles are evidence of this research. The Research Topic presented here, entitled “*Skateboarding and Society: Intersections, Influences, and Implications,*” contributes to this body of literature by demonstrating how skateboarding can be a useful mechanism for negotiating power, placemaking, urban and social development, education, and change.

This special issue of Frontiers in Sports and Active Living, part of the section on the History, Culture, and Sociology of Sports, explores the social scientific dimensions of skateboarding. The seven articles of the special issue (four original research papers, two brief research reports, and one perspective) shed light on the history, culture, challenges, and contributions of skateboarding.

Langseth and Bergsgard provided a reminder that the journey of skateboarding has not always been smooth, exploring a nationwide ban on skateboarding in Norway that existed from 1977 to 1989. Peets et al., exploring contemporary skateboarding among college students, found that conflicts over the use of space between skateboarders and institutions are still a challenge experienced today. That said, instances of more harmonious relationships between skateboarders and their surrounding communities also exist. Book described the case of Malmö, Sweden, as a city that has embraced skateboarding, becoming a recognized skateboarding destination in the process. Book, in addition to Kilberth, outlined strategies of what supportive and collaborative relationships between municipalities and skateboarders/skateboarding may look like and how they can be successful.

Expanding beyond urban development, Peets et al., Glenney et al., Petrone, and Petrone and Beal demonstrated how skateboarding also supports personal and social development. Peets et al. highlighted the community and identity that skateboarders form with each other and how this can enrich university environments. Likewise, Glenney et al. articulated how skateboarding enriches cities by improving human life and wellbeing within them. Similar to Kilberth's typology of skateboarding spaces, Glenney et al. identified skateparks, DIY skateparks, and skate spots as categories of skateboarding spaces that encourage different forms of play that reflect varying relationships with labor, leisure, obedience, and deviance. They proposed that skateboarding offers cities a “surplus value” through “uncommon play” that appreciates labor beyond its exchange value. Instead, they argued that skateboarding offers a form of unalienated labor that provides fulfillment, relationships, and a sense of self in return, ultimately making urban gray spaces more salubrious (p.10).

Petrone posited that urban spaces are positioned as adult spaces, rendering present youth detrimental, undesirable, or at-risk. Therefore, youth-associated activities such as skateboarding are regulated (as demonstrated by the risk management narratives in Langseth and Bergsgard's analysis of Norway's skateboarding ban). However, Petrone argues, such regulations may compromise young people's developmental opportunities and experiences. As such, the author critiques dominant narratives of youth and young people as deficient and requiring adult control, and poses questions to reconsider why/how skateboarding is regulated and to what ends. However, he also acknowledges that “unregulated spaces privilege participants whose identities most closely align with dominant social structures” (p. 2). Thus, he highlights a tension between the positive functions of a participant-driven ethos and the exclusionary systems that can emerge without regulatory intervention.

Petrone and Beal offered a possible response to this tension by highlighting the complementary paradoxical relationship between inclusion and exclusion. They investigated how three skateboarding organizations (Anyone Can Skate, Skate in School, and Skate Center) endeavor to improve diversity, equity, inclusion, and justice within (and beyond) skateboarding. Each organization “establishes pockets of *ex*clusivity” for “demographically similar participants” so they can develop skills, knowledge, and confidence in a comfortable 'safe' space before applying those skills in integrated “brave” spaces outside of the organization—such as skateparks, school, family, etc. (p. 3). In other words, they used strategic exclusion to empower participants to identify and challenge power structures that generate barriers and inequalities so that they may, in turn, promote inclusion. As such, their focus was not just on access, but transformation.

Together, these papers emphasize the creativity, resilience, and self-determination of skateboarders and how skateboarding offers valuable insights into how we relate to space and people. They indicate the ongoing legitimization of skateboarding as it gains popularity, skateboarders continue to advocate for themselves, and institutions increasingly recognize the positive contributions of skateboarding. As such, these papers also highlight the pressing need to understand how skateboarders work within—and outside of—formalized/institutionalized systems, as well as how they create their own organizational frameworks.

A better understanding of urban bodily practices and belonging means considering a diversity of approaches and finding new paths for 21st-century citizenship. The papers in this collection outline how skateboarders' efforts to find and create such alternative paths help reimagine how we take up, share, and use space in ways that can amplify respect and fulfillment, while mitigating conflict. As such, we believe that this special issue is a significant contribution to the advancement of the study of skateboarding. Interested readers now have open access to all articles in the Research Topic. The editors (Bethany Geckle, Kevin Fang, Jorge Saraví and Dax D'Orazio) thank all the authors for their contributions.

